# Effects of Caffeine and Chlorogenic Acid on Nonalcoholic Steatohepatitis in Mice Induced by Choline-Deficient, L-Amino Acid-Defined, High-Fat Diet

**DOI:** 10.3390/nu12123886

**Published:** 2020-12-18

**Authors:** Erdenetsogt Dungubat, Shiori Watabe, Arisa Togashi-Kumagai, Masato Watanabe, Yasuyuki Kobayashi, Naoki Harada, Ryoichi Yamaji, Toshio Fukusato, Galtsog Lodon, Badamjav Sevjid, Yoshihisa Takahashi

**Affiliations:** 1 Department of Pathology, School of Medicine, International University of Health and Welfare, Narita, Chiba 286-8686, Japan; erdenetsogt.dungubat@iuhw.ac.jp; 2 Department of Pathology, School of Biomedicine, Mongolian National University of Medical Sciences, Ulaanbaatar 14210, Mongolia; 3 Department of Pathology, Teikyo University School of Medicine, Tokyo 173-8605, Japan; s.watabe@med.teikyo-u.ac.jp (S.W.); alisakumagai@yahoo.co.jp (A.T.-K.); nabe@med.teikyo-u.ac.jp (M.W.); 4 Division of Applied Life Sciences, Graduate School of Life and Environmental Sciences, Osaka Prefecture University, Sakai, Osaka 599-8531, Japan; sxc02024@edu.osakafu-u.ac.jp (Y.K.); harada@biochem.osakafu-u.ac.jp (N.H.); yamaji@biochem.osakafu-u.ac.jp (R.Y.); 5 General Medical Education and Research Center, Teikyo University, Tokyo 173-8605, Japan; fukusato@med.teikyo-u.ac.jp; 6 Department of Pathology, School of Medicine, Ach Medical University, Ulaanbaatar 18080, Mongolia; lodongaltsog@gmail.com; 7 Department of Gastroenterology, School of Medicine, Mongolian National University of Medical Sciences, Ulaanbaatar 14210, Mongolia; badamjav@mnums.edu.mn

**Keywords:** nonalcoholic steatohepatitis, coffee, caffeine, chlorogenic acid, choline-deficient, L-amino acid-defined, high-fat diet

## Abstract

Several recent experimental studies have investigated the effects of caffeine and chlorogenic acid (CGA), representative ingredients of coffee, on nonalcoholic fatty liver disease (NAFLD)/nonalcoholic steatohepatitis (NASH). However, the results are conflicting, and their effects are yet to be clarified. In the present study, we examined the effects of caffeine and CGA on choline-deficient, L-amino acid-defined, high-fat diet (CDAHFD)-fed mice, relatively new model mice of NASH. Seven-week-old male C57BL/6J mice were divided into the following groups: Control diet (control), CDAHFD (CDAHFD), CDAHFD supplemented with 0.05% (*w*/*w*) caffeine (caffeine), and CDAHFD supplemented with 0.1% (*w*/*w*) CGA (CGA). After seven weeks, the mice were killed and serum biochemical, histopathological, and molecular analyses were performed. Serum alanine aminotransferase (ALT) levels were significantly higher in the caffeine and CGA groups than in the CDAHFD group. On image analysis, the prevalence of Oil red O-positive areas (reflecting steatosis) was significantly higher in the caffeine group than in the CDAHFD group, and that of CD45R-positive areas (reflecting lymphocytic infiltration) in the hepatic lobule was significantly higher in the caffeine and CGA groups than in the CDAHFD group. Hepatic expression of *interleukin (IL)-6* mRNA was higher in the caffeine and CGA groups than in the CDAHFD group, and the difference was statistically significant for the caffeine group. In conclusion, in the present study, caffeine and CGA significantly worsened the markers of liver cell injury, inflammation, and/or steatosis in NASH lesions in mice.

## 1. Introduction

Nonalcoholic fatty liver disease (NAFLD) is defined as accumulation of excessive fat in the hepatocytes of patients without a history of large amount of alcohol drinking. NAFLD includes a spectrum of liver diseases that ranges from simple steatosis (nonalcoholic fatty liver) to nonalcoholic steatohepatitis (NASH), which can progress to liver cirrhosis and hepatocellular carcinoma (HCC) [1,2,3]. NAFLD/NASH is rapidly increasing worldwide, in line with the increased prevalence of obesity because of lifestyle changes in recent years with the consumption of energy-rich diets. The prevalence of NAFLD in the general population of the world was estimated to be 25%, and the prevalence of NASH in the world was estimated to be 3–5% [4,5,6,7], demonstrating the importance of developing effective methods for their prevention and treatment.

Coffee is a brewed drink prepared from roasted coffee beans and is one of the most frequently consumed drinks in the world. Chemically, coffee contains many physiologically active compounds [8,9]. Coffee consumption is associated with reduced risk of several diseases including type 2 diabetes, Parkinson’s disease, and HCC [10,11,12]. Previous clinical and experimental studies also suggested inhibitory effects of coffee on NAFLD/NASH [13,14,15]; however, the effective ingredients and mechanisms have not been determined.

Caffeine and chlorogenic acid (CGA) are representative ingredients of coffee, and the contents of caffeine and CGA in 200–240 mL of coffee are estimated to be 72–130 mg and 70–350 mg, respectively [16]. Caffeine is a xanthine alkaloid that is considered to have energy expending, antioxidant, and anti-inflammatory activities [17,18,19,20]. CGA is a polyphenol that is known to have antioxidant, hepatoprotective, anti-inflammatory, and anti-obesity effects [21]. Several recent experimental studies have investigated the effects of caffeine and CGA on NAFLD/NASH under the assumption that they might underlie the effects of coffee on NAFLD/NASH [19,22,23,24,25,26,27,28,29,30]. Although most of those previous studies showed the inhibitory effects of caffeine and CGA on NAFLD/NASH [19,22,23,24,25,27,28,29], several studies have also reported exacerbating effects [26,30]. Thus, the effects of these ingredients should be clarified.

Here, we examined the effects of caffeine and CGA on the hepatic lesions of choline-deficient, L-amino acid-defined, high-fat diet (CDAHFD)-fed mice, a relatively new animal model of NASH that was developed to improve methionine- and choline-deficient (MCD) and high-fat (HF) diet models. We applied the CDAHFD model in this study because Matsumoto et al. [31] reported that C57BL/6J mice that were fed the diet maintained or gained weight and developed NASH with fibrosis in only six weeks.

## 2. Materials and Methods

### 2.1. Mice and Experimental Protocol

Twenty-four 6-week-old male C57BL/6J mice were purchased from CLEA Japan (Tokyo, Japan). Mice were acclimated to the environment for one week, and then randomly divided into the following groups (*n* = 6, each): Control, CDAHFD, caffeine, and CGA. Mice in the control group were fed a control diet (CRF-1) (Oriental Yeast, Tokyo, Japan); the CDAHFD group was fed CDAHFD (A06071302) (Research Diets, New Brunswick, NJ, USA); the caffeine group was fed CDAHFD supplemented with 0.05% (*w*/*w*) caffeine; and the CGA group was fed CDAHFD supplemented with 0.1% (*w*/*w*) CGA, ad libitum. Caffeine and CGA were purchased from the FUJIFILM Wako Pure Chemical Corporation (Osaka, Japan). These compounds were mixed in the CDAHFD at the above-mentioned concentrations. The feeds were stored at 4 °C. The feeds were exchanged three times/week, and food intake was recorded. Food consumption and calorie intake per day were calculated each time during replacement of diet. The experiment was performed at the Animal Center of Teikyo University School of Medicine (Tokyo, Japan). The conditions were as follows: 25 °C, 45% humidity, and a 12-h light/12-h dark cycle. Seven weeks later, the mice were killed by decapitation under deep anesthesia. A blood sample from each mouse was collected at decapitation, and the serum was separated via centrifugation. The liver of each mouse was extirpated and weighed, and samples for histological analysis, RNA purification, and snap-freezing were collected. The epididymal adipose tissue (EAT) was also excised and weighed to use as an indicator of the amount of visceral fat.

This study was performed in conformity to the Guide for the Care and Use of Laboratory Animals of the National Institutes of Health. The experimental protocol was approved by the Ethics Committee of Teikyo University (on 25 February 2016; Permit No. 15-047). All animals received humane care, and all efforts were made to alleviate suffering.

### 2.2. Assessment of Serum Biochemical Parameters

Serum alanine aminotransferase (ALT), aspartate aminotransferase (AST), triglyceride (TG), total cholesterol (T-Cho), glucose, insulin, and adiponectin levels were measured as described in our previous studies [32,33].

### 2.3. Histopathological Analysis

The central parts of the left lateral lobe and medial lobe of the liver were fixed in 10% neutral buffered formalin and routinely processed for light microscopy. Hematoxylin and eosin (H&E) staining and Sirius red staining were performed to evaluate hepatic histopathology including fibrosis. Oil red O staining was also performed using frozen liver tissue sections to evaluate hepatic steatosis. The histopathology was evaluated semi-quantitatively using the validated scoring system by Kleiner et al. [34]. Detailed scoring criteria for steatosis, lobular and portal inflammation, hepatocellular ballooning, and perisinusoidal fibrosis are described in our previous papers [32,33]. The method of calculation of NAFLD activity score (NAS) and staging of fibrosis are also described in our previous papers [32,33].

The histological slides were observed by three hepatic pathologists (E.D., T.F., and Y.T.). Generally, each pathologist’s evaluation showed good concordance. When there were minor differences in evaluation, the three pathologists had a discussion and made the final decision.

### 2.4. Immunohistochemistry

Immunohistochemical staining for CD45R and α-smooth muscle actin (α-SMA) was performed to detect infiltrating lymphocytes and activated hepatic stellate cells (HSCs), respectively. Paraffin sections of liver specimens were immunostained with anti-mouse/human CD45R monoclonal antibody (catalog No. 103202; clone: RA3-6B2; dilution: 1:100; BioLegend, San Diego, CA, USA) using the Histofine Simple Stain Mouse MAX-PO (Rat) kit (Nichirei Biosciences, Tokyo, Japan), and with anti-α-SMA monoclonal antibody (catalog No. M0851; clone: 1A4; dilution: 1:100; Agilent Technologies, Santa Clara, CA, USA) using the Histofine Mousestain kit (Nichirei Biosciences), respectively, following instructions of the manufacturers. The method of antigen retrieval was heating the sections for 20–40 min in citrate buffer (pH 6.0) in a water bath.

### 2.5. Image Analysis of Histological Slides

We performed image analysis to objectively assess the degree of steatosis and fibrosis, and the number of lymphocytes and activated HSCs. Photomicrographs of four randomly selected intralobular fields were obtained for each Sirius red, α-SMA, and CD45R staining slide (magnification: 400× for Sirius red staining, 400× for α-SMA staining, and 200× for CD45R staining). Photomicrographs of two randomly selected 100× fields were obtained for each Oil red O staining slide. Several photomicrographs were taken for each mouse to minimize the bias by selection of the microscopic field; all these photomicrographs were used for image analysis as described in our previous studies [32,33]. The prevalence of Oil red O-, Sirius red-, CD45R-, and α-SMA-positive areas in these photomicrographs was analyzed using the image analysis software WinROOF (Mitani Corporation, Fukui, Japan).

### 2.6. Hepatic Levels of TG and T-Cho

We measured TG and T-Cho levels in the liver biochemically using frozen tissues. The detailed methods are described in our previous paper [33].

### 2.7. 4-Hydroxynonenal (4-HNE) Levels in the Liver

The hepatic 4-HNE levels were measured to evaluate the oxidative injury to lipids in the liver as described in our previous study [33].

### 2.8. Real-Time Reverse Transcription Polymerase Chain Reaction (RT-PCR) Analysis

To examine the molecular mechanisms by which caffeine and CGA influence the NASH lesion in mice, we measured mRNA expression of cytokine and receptor genes in the liver by real-time RT-PCR. The expression of *tumor necrosis factor (TNF)-α*, *interleukin (IL)-6*, *IL-10*, *peroxisome proliferator-activated receptor (PPAR)γ*, and *transforming growth factor (TGF)-β1* genes was measured and the *TATA-box binding protein (TBP)* gene was used as the internal control. The detailed methods and primer sequences of the genes, other than *IL-10*, are described in our previous papers [32,33]. The primers for the *IL-10* gene were as follows: forward, 5′-GCGCTGTCATCGATTTCTCC-3′; reverse, 5′-CATGGCCTTGTAGACACCTTGG-3′.

Additionally, we performed real-time RT-PCR to evaluate the mRNA expression of genes involved in lipidic and glucidic metabolism in the liver. The expression of *CD36*, *PPARα*, *microsome triglyceride transfer protein (MTP)*, *carnitine palmitoyltransferase (CPT) 1A*, *sterol regulatory element-binding protein (SREBP)-1c*, *fatty acid synthase (FASN)*, *acetyl-CoA carboxylase (ACC)*, *phosphoenolpyruvate carboxykinase (PEPCK)*, and *pyruvate carboxylase (PC)* genes was measured and the *TBP* gene was used as the internal control. The methods are the same as additional real-time RT-PCR in our previous study [33], and the primers for each gene are shown in Table 1.

### 2.9. Statistical Analysis

For continuous variables, data are presented as means ± standard deviation (SD). One-way analysis of variance (ANOVA) followed by Dunnett’s post-hoc test was performed to evaluate the significance of the differences. For semi-quantitative data in histological evaluation, data are presented in terms of the median (min. to max.). The Kruskal-Wallis test followed by Steel’s post-hoc test was performed to determine statistical significance. Statistical significance was set at a *p*-value < 0.05.

## 3. Results

### 3.1. General Findings

No mice died during the experiment. Table 2 shows data of food consumption, calorie intake, and body and organ weight of the mice. Food consumption was significantly lower (*p* < 0.001, *p* = 0.006, and *p* < 0.001, respectively), whereas calorie intake was significantly higher (*p* = 0.001, *p* < 0.001, and *p* = 0.001, respectively), in the CDAHFD, caffeine, and CGA groups than in the control group. Food consumption and calorie intake were significantly higher in the caffeine group than in the CDAHFD group (both *p* < 0.001). Body weight was significantly lower in the CDAHFD, caffeine, and CGA groups than in the control group (*p* < 0.001 for all) but was not significantly influenced by caffeine or CGA. Liver weight was significantly higher in the CDAHFD, caffeine, and CGA groups than in the control group (*p* = 0.003, *p* = 0.007, and *p* < 0.001, respectively). The liver/body weight ratios were significantly higher in the CDAHFD, caffeine, and CGA groups than in the control group (*p* < 0.001 for all). However, caffeine or CGA did not significantly influence these parameters. The EAT weight was significantly lower in the CDAHFD, caffeine, and CGA groups than in the control group (*p* < 0.001 for all), and the EAT/body weight ratios were significantly lower in the CDAHFD, caffeine, and CGA groups than in the control group (*p* = 0.001, *p* < 0.001, and *p* = 0.006, respectively). However, caffeine or CGA did not significantly influence these parameters.

### 3.2. Biochemical Data of the Serum

Table 3 shows the serum data of each group. Serum AST and ALT levels were significantly higher in the CDAHFD, caffeine, and CGA groups than in the control group (*p* < 0.001 for all) and tended to be higher (AST) and significantly higher (ALT; *p* = 0.048 and 0.024, respectively) in the caffeine and CGA groups than in the CDAHFD group. Serum T-Cho (*p* < 0.001 for all) and TG (*p* = 0.004, 0.001, and 0.014, respectively) levels were significantly lower in the CDAHFD, caffeine, and CGA groups than in the control group. Serum insulin levels were significantly lower in the CDAHFD and CGA groups than in the control group (*p* = 0.012 and 0.008, respectively). Serum adiponectin levels were significantly lower in the CDAHFD, caffeine, and CGA groups than in the control group (*p* = 0.006, 0.008, and 0.005, respectively). Neither caffeine nor CGA significantly influenced these parameters. However, serum insulin levels tended to be higher in the caffeine group than in the CDAHFD group. Serum glucose levels were significantly higher in the caffeine group than in the control group (*p* = 0.022).

### 3.3. Histopathological Findings

**Figure 1:**
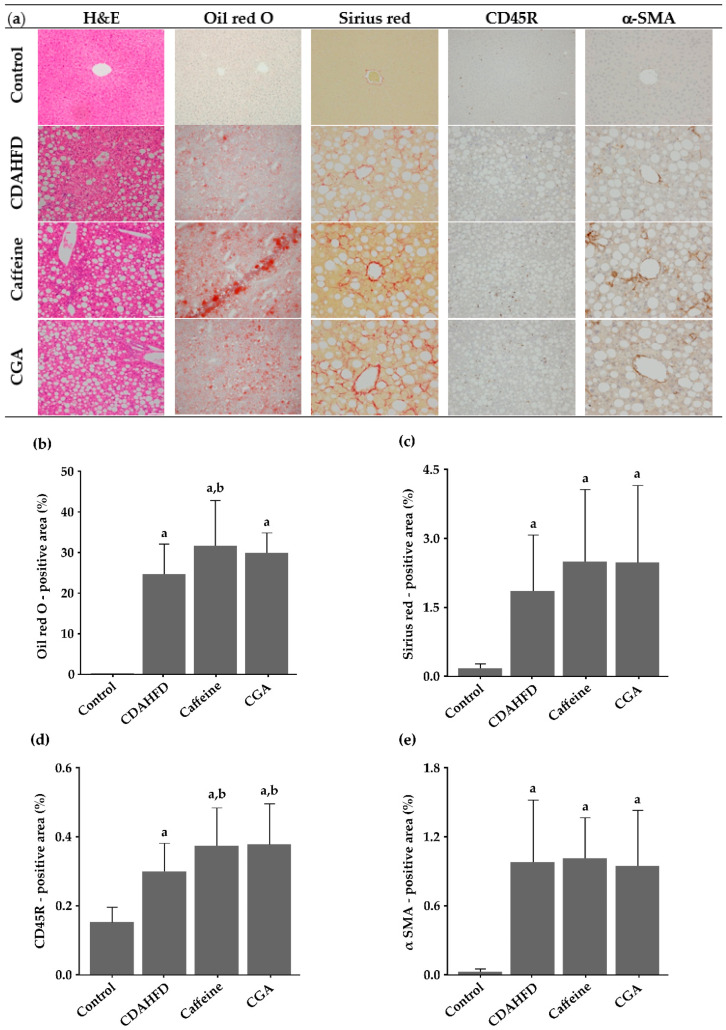
Photomicrographs of the liver (**a**) and results of quantitative image analysis (**b**–**e**). (**a**) The mice in the choline-deficient, L-amino acid-defined, high-fat diet (CDAHFD), caffeine, and chlorogenic acid (CGA) groups show liver histopathological features of nonalcoholic steatohepatitis (NASH). H&E, Hematoxylin and eosin. (**b**) The prevalence of Oil red O-positive areas reflecting the degree of steatosis, is significantly higher in the CDAHFD, caffeine, and CGA groups than in the control group; it is higher in the caffeine and CGA groups than in the CDAHFD group, and the difference is statistically significant between the caffeine and CDAHFD groups. (**c**) The prevalence of Sirius red-positive areas, that reflects the degree of fibrosis, in the hepatic lobule, is significantly higher in the CDAHFD, caffeine, and CGA groups than in the control group, and tends to be higher in the caffeine and CGA groups than in the CDAHFD group. (**d**) The prevalence of CD45R-positive areas in the hepatic lobule, that reflects the degree of intralobular lymphocytic infiltration, is significantly higher in the CDAHFD, caffeine, and CGA groups than in the control group and significantly higher in the caffeine and CGA groups than in the CDAHFD group. (**e**) The prevalence of α-SMA-positive areas in the hepatic lobule, that reflects the number of activated hepatic stellate cells (HSCs), is significantly higher in the CDAHFD, caffeine, and CGA groups than in the control group, but caffeine or CGA does not significantly influence it. ^a^ Significantly different from the control group (*p* < 0.05). ^b^ Significantly different from the CDAHFD group (*p* < 0.05).

The mice in the CDAHFD, caffeine, and CGA groups showed liver histopathological features of NASH, although mice in the control group did not (Figure 1a). Table 4 shows the semi-quantitative histological findings of each group. Steatosis and intralobular inflammation (both *p* = 0.001 for all), as well as ballooning (*p* = 0.002 for all), were significantly more severe in the CDAHFD, caffeine, and CGA groups than in the control group. Perisinusoidal fibrosis was significantly more severe in the CDAHFD, caffeine, and CGA groups than in the control group (*p* = 0.002, 0.004, and 0.002, respectively). However, neither caffeine nor CGA significantly influenced these parameters. NAS (*p* = 0.002 for all) and fibrosis stage (*p* = 0.007 for all) were significantly higher in the CDAHFD, caffeine, and CGA groups than in the control group. However, caffeine or CGA did not significantly influence them. Portal inflammation was not significantly different among the groups.

### 3.4. Image Analysis of Histological Slides

Figure 1b–e shows the results of the quantitative image analysis. The prevalence of Oil red O-positive areas, that reflects the degree of steatosis, was significantly higher in the CDAHFD, caffeine, and CGA groups than in the control group (*p* < 0.001 for all). It was higher in the caffeine and CGA groups than in the CDAHFD group, and the difference was statistically significant between the caffeine and CDAHFD groups (*p* = 0.036). The prevalence of Sirius red-positive areas, that reflects the degree of fibrosis, in the hepatic lobule, was significantly higher in the CDAHFD, caffeine, and CGA groups than in the control group (*p* < 0.001 for all). It tended to be higher in the caffeine and CGA groups than in the CDAHFD group, but the differences were not statistically significant. The prevalence of CD45R-positive areas in the hepatic lobule, that reflects the degree of intralobular lymphocytic infiltration, was significantly higher in the CDAHFD, caffeine, and CGA groups than in the control group (*p* < 0.001 for all), and significantly higher in the caffeine and CGA groups than in the CDAHFD group (*p* = 0.008 and 0.006, respectively). The prevalence of α-SMA-positive areas in the hepatic lobule, that reflects the number of activated HSCs, was significantly higher in the CDAHFD, caffeine, and CGA groups than in the control group (*p* < 0.001 for all) but was not significantly influenced by caffeine or CGA.

### 3.5. TG and T-Cho Levels in the Liver

**Figure 2:**
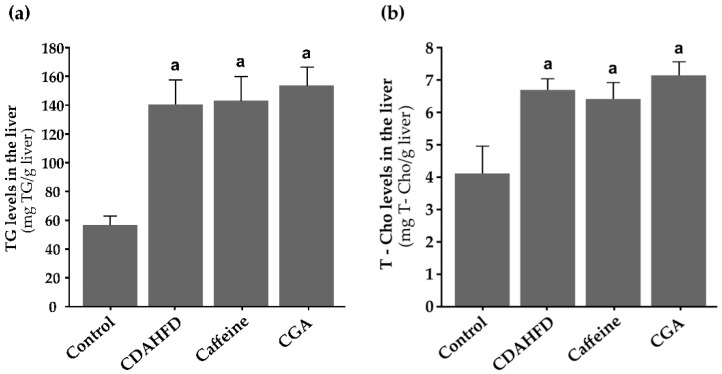
Triglyceride (TG) and total cholesterol (T-Cho) levels in the liver (**a**,**b**). (**a**) TG levels in the liver are significantly higher in the CDAHFD, caffeine, and CGA groups than in the control group and tend to be higher in the caffeine and CGA groups than in the CDAHFD group. (**b**) T-Cho levels in the liver are significantly higher in the CDAHFD, caffeine, and CGA groups than in the control group, but caffeine or CGA does not significantly influence them. Generally, hepatic TG levels are 15–20-fold higher than hepatic T-Cho levels. ^a^ Significantly different from the control group (*p* < 0.05).

Figure 2a,b shows TG and T-Cho levels in the liver. TG levels in the liver were significantly higher in the CDAHFD, caffeine, and CGA groups than in the control group (*p* < 0.001 for all) and tended to be higher in the caffeine and CGA groups than in the CDAHFD group. Hepatic T-Cho levels were significantly higher in the CDAHFD, caffeine, and CGA groups than in the control group (*p* < 0.001 for all), but neither caffeine nor CGA significantly influenced them. Generally, hepatic TG levels were 15–20-fold higher than hepatic T-Cho levels.

### 3.6. Hepatic 4-HNE Levels

**Figure 3:**
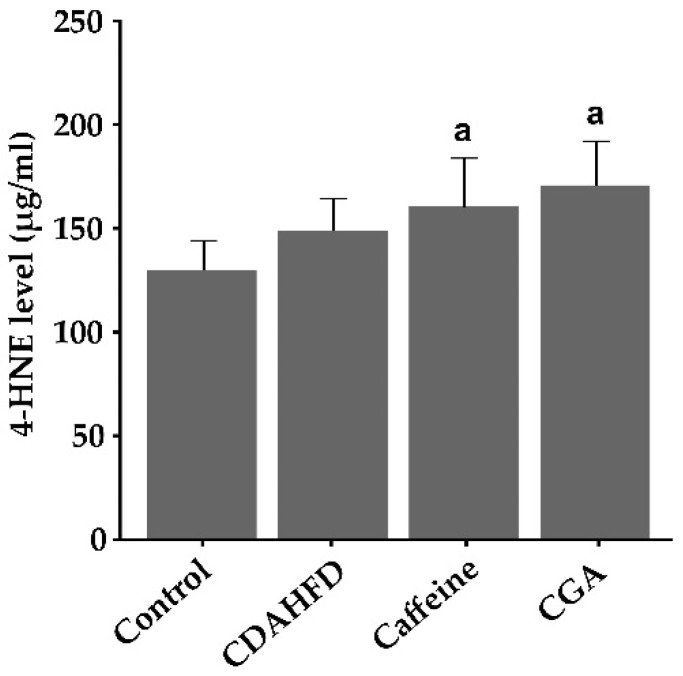
4-Hydroxynonenal (4-HNE) levels in the liver. Levels are significantly higher in the caffeine and CGA groups than in the control group but are not significantly different from those in the CDAHFD group. ^a^ Significantly different from the control group (*p* < 0.05).

Hepatic 4-HNE levels were significantly higher in the caffeine and CGA groups than in the control group (*p* = 0.013 and 0.002, respectively), but were not significantly different from those in the CDAHFD group (Figure 3).

### 3.7. Gene Expression in the Hepatic Tissue

**Figure 4:**
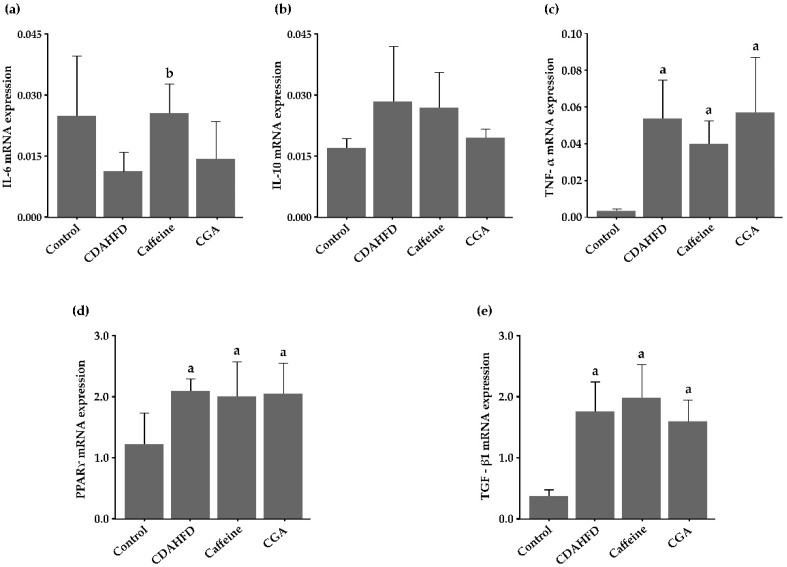
Hepatic expression of cytokine and receptor genes determined by real-time RT-PCR (**a**–**e**). (**a**) The hepatic *IL-6* expression is higher in the caffeine and CGA groups than in the CDAHFD group, and the difference is statistically significant between the caffeine and CDAHFD groups. (**b**) Hepatic expression of *IL-10* mRNA is not significantly different among the experimental groups. (**c**–**e**) Levels of *tumor necrosis factor (TNF)-α*, *peroxisome proliferator-activated receptor (PPAR)γ*, and *transforming growth factor (TGF)-β1* mRNA expression in the liver are significantly higher in the CDAHFD, caffeine, and CGA groups than in the control group. However, these are not significantly influenced by either caffeine or CGA. ^a^ Significantly different from the control group (*p* < 0.05). ^b^ Significantly different from the CDAHFD group (*p* < 0.05).

Figure 4a–e shows the expression of hepatic cytokine and receptor genes that was determined by real-time RT-PCR. The hepatic *IL-6* expression was lower in the CDAHFD group than in the control group; it was higher in the caffeine and CGA groups than in the CDAHFD group, and the difference was statistically significant between the caffeine and CDAHFD groups (*p* = 0.043). Hepatic levels of *TNF-α* (*p* < 0.001, *p* = 0.008, and *p* < 0.001, respectively), *PPARγ* (*p* = 0.013, 0.025, and 0.017, respectively), and *TGF-β1* (*p* < 0.001 for all) mRNA expression were significantly higher in the CDAHFD, caffeine, and CGA groups than in the control group. However, these were not significantly influenced by either caffeine or CGA. Hepatic expression of *IL-10* mRNA was not significantly different among the experimental groups.

**Figure 5:**
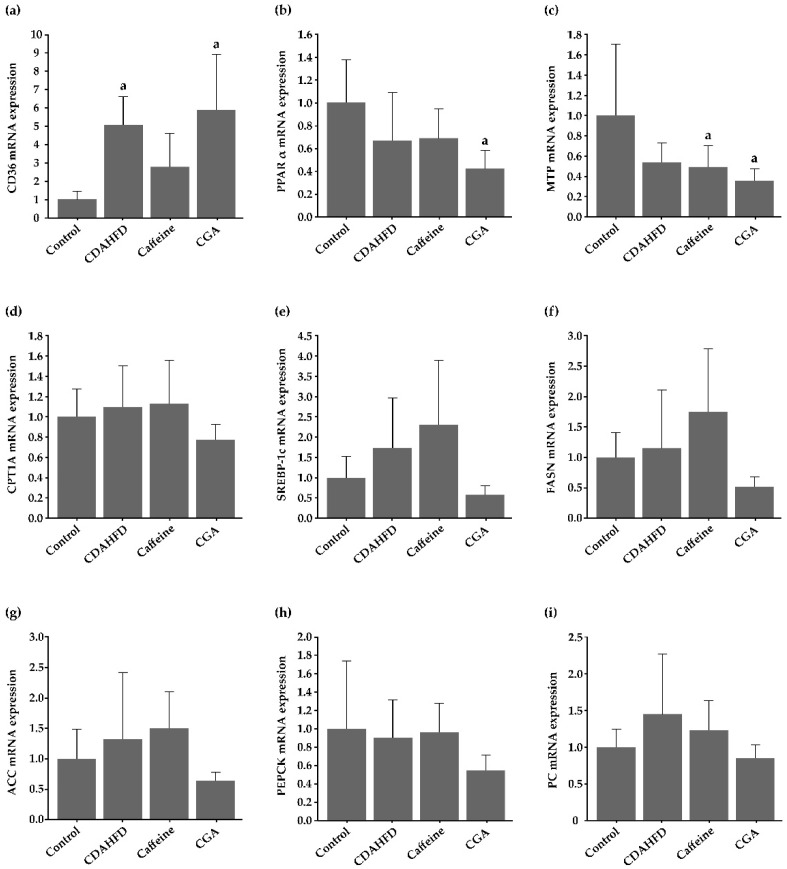
Expression of genes that are associated with lipidic and glucidic metabolism in the liver, determined by real-time RT-PCR (**a**–**i**). (**a**) Significantly more *CD36* is expressed in the livers of the CDAHFD and CGA groups than the control group and are not significantly influenced by caffein or CGA. (**b**) Expression of *PPARα* in the liver is significantly lower in the CGA group than in the control group, but not significantly different from the CDAHFD group. (**c**) *Microsome triglyceride transfer protein (MTP)* expression in the liver is significantly lower in the caffeine and CGA groups than in the control group, but not significantly different from the CDAHFD group. (**d**–**i**) Hepatic expression of *carnitine palmitoyltransferase (CPT) 1A*, *sterol regulatory element-binding protein (SREBP)-1c*, *fatty acid synthase (FASN)*, *acetyl-CoA carboxylase (ACC)*, *phosphoenolpyruvate carboxykinase (PEPCK)*, and *pyruvate carboxylase (PC)* mRNA is not significantly different among the experimental groups. ^a^ Significantly different from the control group (*p* < 0.05).

Figure 5a–i shows the expression of genes that are involved in lipidic and glucidic metabolism in the liver, that was determined by additional real-time RT-PCR. Significantly more *CD36* was expressed in the livers of the CDAHFD and CGA groups than the control group (*p* = 0.002 and *p* < 0.001, respectively), and were not significantly influenced by caffeine or CGA. Hepatic expression of *PPARα* was significantly lower in the CGA group than in the control group (*p* = 0.007), but not significantly different from the CDAHFD group. Hepatic *MTP* expression was significantly lower in the caffeine and CGA groups than in the control group (*p* = 0.044 and 0.013, respectively), but not significantly different from the CDAHFD group. Expression of *PPARα* and *MTP* tended to be lower in the CDAHFD group than in the control group. Hepatic expression of *CPT 1A*, *SREBP-1c*, *FASN*, *ACC*, *PEPCK*, and *PC* mRNA was not significantly different among the experimental groups.

## 4. Discussion

In this study, caffeine and CGA administration to a NASH mouse model significantly increased serum ALT levels (a liver cell injury marker) and CD45R-positive area in the hepatic lobule (that reflects intralobular lymphocytic infiltration). The Oil red O-positive area in the liver (reflecting hepatic steatosis) was also increased, and the effect was statistically significant for caffeine. Therefore, in the present study, caffeine and CGA significantly worsened the markers of liver cell injury, inflammation, and/or steatosis in NASH in mice. Semi-quantitative histopathological findings showed that neither caffeine nor CGA significantly influenced steatosis and intralobular inflammation. However, we induced advanced steatosis and inflammation using CDAHFD, and all mice in the CDAHFD, caffeine, and CGA groups were evaluated as grade 3 for steatosis and intralobular inflammation. Therefore, these parameters were difficult to accurately evaluate using Kleiner’s scoring system. Sirius red- and α-SMA-positive areas, biochemically determined hepatic TG and T-Cho levels, and the expression genes other than *IL-6* in the liver were not significantly influenced by caffeine or CGA. However, a single agent rarely significantly influences all NASH parameters. For example, we previously found that consuming small quantities of ethanol significantly improves serum ALT and AST values, and intralobular and portal inflammation, but does not significantly influence steatosis, fibrosis, insulin and adiponectin in the serum, and hepatic 4-HNE levels, or cytokine and receptor gene expression in the liver [33].

Protective effects of coffee against NAFLD/NASH have been suggested in previous clinical and experimental studies [13,14,15], and the effects of caffeine and CGA on NAFLD/NASH have been examined in recent years under the assumption that they might be the effective ingredients. In most previous experimental studies on the effects of caffeine on NAFLD/NASH, caffeine exhibited inhibitory effects [19,22,23,24,25]. However, Hu et al. [26] reported that prenatal caffeine exposure increased susceptibility to NAFLD in female offspring rats. Conflicting results were also reported in human studies. Birerdinc et al. [35] reported that caffeine was protective in NAFLD patients, but Shen et al. [36] reported that total caffeine intake was not associated with the prevalence or hepatic fibrosis in NAFLD. Thus, the effects of caffeine on NAFLD/NASH have not yet been clarified. In this study, it was suggested that caffeine might actually exacerbate NASH in mice. In most previous experimental studies on the effects of CGA on NAFLD/NASH, CGA exhibited inhibitory effects [27,28,29]; however, Mubarak et al. [30] reported that supplementation of an HF diet with CGA caused insulin resistance and hepatic lipid accumulation in mice. Here, as in the study by Mubarak et al., it was suggested that CGA might exacerbate NAFLD/NASH in mice. In the present study, hepatic *MTP* expression was lower and hepatic 4-HNE and serum ALT levels were higher in the caffeine and CGA groups than in the control and CDAHFD groups. As the inhibition of MTP is responsible for oxidative stress and ALT elevation [37], a decreased expression of *MTP* by caffeine and CGA might be associated with liver cell injury. The results of this study cannot attribute the inhibitory effects of coffee on NAFLD/NASH to caffeine or CGA, and other ingredients of coffee might exert inhibitory effects. It was recently reported that consumption of decaffeinated coffee protected against the development of early NASH in mice [38].

The difference in experimental conditions is conceivably the cause of conflicting results in studies on the effects of caffeine and CGA on NAFLD/NASH. Animal models of NAFLD/NASH used in these studies were varied, including nutritional models such as HF, high-cholesterol, and high-fructose diets, and genetic models such as KK-Ay mice. Various animals, including C57BL/6 mice, Sprague-Dawley rats, and Wistar rats, were used in the nutritional models. Furthermore, the method of administration was varied, including drinking water, diet, oral gavage, and intragastric; the dosage was also different in each study. Here, we used C57BL/6J mice fed CDAHFD as an animal model of NASH. C57BL/6J mouse is one of the most commonly used mouse strains and is frequently used in studies of NAFLD/NASH. We administered caffeine and CGA by mixing them in the diet, since it is a commonly used method to administer caffeine and CGA to experimental animals, and the easiest to perform. We applied 0.05% (*w*/*w*) of caffeine and 0.1% (*w*/*w*) of CGA because they are standard doses with proven positive or negative effects in experimental animals [30,39,40,41,42,43]. Caffeine inhibited fat accumulation in ICR mice at the same dose and the same method as in the present study [40]. Mubarak et al. [30] administered CGA at the same dose and in the same manner as in the present study. Daily caffeine and CGA intake were 1.35 (65 mg/kg) and 2.3 (112 mg/kg) mg, respectively, in this study. Therefore, the caffeine and CGA intake per body weight in this study was about 20-fold higher than that with human coffee intake; thus, the results cannot be simply extrapolated to humans.

CDAHFD is a relatively new model diet of NASH. Classic nutritional models of NASH include the MCD and HF diet models. Although the MCD diet induces severe histopathology of NASH, the metabolic status of the model is opposite to that of human NASH with a remarkable decrease in body weight. Although the metabolic status of the HF diet model is similar to that of human NAFLD/NASH, histopathological changes in the model are mild [44]. The CDAHFD was developed to overcome the shortcomings of these models. It was reported that C57BL/6J mice fed CDAHFD maintained or gained weight and developed fatty liver with fibrosis by week 6 [31]. In the present study, CDAHFD also induced severe NASH with fibrosis in C57BL/6J mice in seven weeks. However, body and EAT weight, and serum levels of TG, T-Cho, and insulin in the CDAHFD group were significantly lower than those in the control group. Thus, the metabolic status of the present model was thought to be opposite to that of human NAFLD/NASH. In addition, CDAHFD did not significantly influence the expression of genes associated with lipidic and glucidic metabolism except for *CD36*. This finding suggested that this model might not be appropriate to evaluate molecular mechanisms of lipidic and glucidic metabolism. The present findings indicated that the CDAHFD model might not be appropriate for analyzing NAFLD/NASH pathophysiology, but it does have the advantage of rapidly inducing histopathological changes in NASH. We also believe that the intensive biochemical, pathological, and molecular analyses of the CDAHFD group in this study generated many new and important findings about the model. For example, CDAHFD did not significantly influence the expression of genes associated with glucidic metabolism (i.e., *PEPCK* and *PC*), whereas it significantly increased the expression of *PPARγ* and *CD36* in the liver. CD36 is a transporter of fatty acid and the target gene of PPARγ, which is induced by HF diet feeding [45]. Therefore, CDAHFD may cause hepatic steatosis by enhancing lipid intake. PPARα regulates MTP (which is associated with TG transport) expression and plays key roles in suppressing NAFLD [46,47]. In the present study, hepatic expression of *PPARα* and *MTP* tended to be lower in the CDAHFD group than in the control group. Therefore, impaired transport of TG might also be associated with CDAHFD-induced hepatic steatosis. In contrast, hepatic expression of genes associated with fatty acid synthesis (i.e., *SREBP-1c*, *FASN*, and *ACC*) and β-oxidation (i.e., *CPT 1A*) did not show remarkable changes by CDAHFD feeding. Review articles of animal models of NAFLD/NASH by our group [44] and other groups [48,49] have emphasized that no animal models of NAFLD/NASH are complete, and to reflect the entire spectrum of NASH in one model is impossible. Many models of severe inflammation such as the MCD diet model, cholesterol and cholate model, and *PTEN* null mice do not develop insulin resistance. Nonetheless, they are considered useful for evaluating NASH histopathology and the effects of various substances on NASH [50,51,52]. Therefore, the present model is thought to be a robust model for analyzing the histopathology of NASH, but it may not be appropriate for analyzing the pathophysiology of the disease. CDAHFD is a relatively novel diet model of NASH, and further characterization of the model (e.g., effects on genetically obese mice) should be performed in the future. Several animal models with similar pathophysiology to human NAFLD/NASH and relatively severe histopathological changes have been developed recently. For example, Kohli et al. [53] reported that mice fed HF diet and drinking water with 55% fructose and 45% sucrose (*w/v*) developed increased body weight, body fat mass, fasting glucose, and were insulin resistant. Histopathologically, those mice showed substantial steatosis with inflammatory changes, and 16% of the mice showed stage 2 fibrosis. This kind of model may be more useful in evaluating the effects of caffeine and CGA on NASH.

Notably, food consumption was significantly higher in the caffeine group than in the CDAHFD group. This was an unexpected result since it is known that caffeine lowers energy intake [54]. However, it is also known that caffeine causes the release of catecholamines and increases energy expenditure [17,20]. In the present study, despite the significantly higher food consumption and calorie intake, the caffeine group did not show increased body, liver, and EAT weight compared with the CDAHFD group. Thus, caffeine might have promoted appetite through activation of metabolism. Here, increased consumption of CDAHFD may be one cause of the exacerbating effects of caffeine on NASH. *IL-6* expression in the liver determined by real-time RT-PCR was higher in the caffeine and CGA groups than in the CDAHFD group, and the difference was statistically significant between the caffeine and CDAHFD groups. However, the hepatic expression of *IL-6* was lower in the CDAHFD group than in the control group. Therefore, caffeine might restore normal *IL-6* expression.

It was interesting that serum insulin levels tended to be higher in the caffeine group than in the CDAHFD group. Since serum insulin levels were significantly lower in the CDAHFD group than in the control group, the data may suggest a protective role of caffeine. However, another hypothesis is that caffeine decreased the insulin sensitivity, since serum glucose levels were higher in the caffeine group than in the CDAHFD group. Actually, studies that observe glucose or insulin activity after acute caffeine consumption show an overall negative effect [55,56,57], and Jarrar et al. [58] reported that caffeine seemed to decrease insulin sensitivity because glucose levels were sustained in spite of high insulin levels.

This study has several limitations. The statistical significance of several parameters was marginal (e.g., serum ALT levels and Oil red O-positive areas) and may not be sufficiently convincing. Furthermore, a large sample size might contribute to statistical significance in image analyses. Actually, there was no significant difference in the hepatic levels of TG (major lipid in the liver) between the caffeine and CDAHFD groups, although significant difference was observed in the prevalence of Oil red O-positive areas. However, sampling bias might be present in the biochemical analysis of TG, since only a small amount of liver tissue (approximately 50 mg for each mouse) was analyzed. As mentioned above, the results of experimental studies on the effects of caffeine and CGA on NAFLD/NASH are conflicting, and conceivably due to differences in experimental conditions. The current model is not common. Instead, the beneficial effects of caffeine and CGA have previously been demonstrated in the common models of NAFLD (e.g., HF diet model). Importantly, most pathophysiological parameters are not correlated with human signs. Therefore, a potential cause of the opposite findings of the present study to most of previous studies may be the peculiarity of the CDAHFD model. In the future, other animal models of NAFLD/NASH (especially models with similar pathophysiology to human disease) and administration methods should be tested. Furthermore, the dose-response relationship should also be further examined by setting multiple dosages.

## 5. Conclusions

In this study, caffeine and CGA significantly worsened the markers of liver cell injury, inflammation, and/or steatosis in NASH lesions in mice. Since conflicting results have been reported for the effects of caffeine and CGA on NAFLD/NASH, further studies including those to clarify the dose-response relationship are recommended in the future.

## Figures and Tables

**Table 1:** Primers used in additional real-time RT-PCR.

Genes	Forward Primers (5′→3′)	Reverse Primers (5′→3′)
*CD36*	GCCAAGCTATTGCGACATGA	CAATGGTTGTCTGGATTCTGG
*PPARα*	AATGCAATTCGCTTTGGAAG	GGCCTTGACCTTGTTCATGT
*MTP*	CATCTCCACAGTGCAGTTCTCACA	GGAGTTCACATCCGGCCACTA
*CPT 1A*	GATCTACAATTCCCCTCTGCTCT	TAGAGCCAGACCTTGAAGTAACG
*SREBP-1c*	TGGACTACTAGTGTTGGCCTGCTT	ATCCAGGTCAGCTTGTTTGCGATG
*FASN*	TGGGTTCTAGCCAGCAGAGT	ACCACCAGAGACCGTTATGC
*ACC*	GGAGATGTACGCTGACCGAGAA	ACCCGACGCATGGTTTTCA
*PEPCK*	GGTGTTTACTGGGAAGGCATC	CAATAAGGGGCACTGGCTG
*PC*	GAGCTTATCCCGAACATCCC	TCCATACCATTCTCTTTGGCC
*TBP*	CTGGAATTGTACCGCAGCTT	ATGATGACTGCAGCAAATCG

*ACC*, acetyl-CoA carboxylase; *CPT*, carnitine palmitoyltransferase; *FASN*, fatty acid synthase; *MTP*, microsome triglyceride transfer protein; *PC*, pyruvate carboxylase; *PEPCK*, phosphoenolpyruvate carboxykinase; *PPAR*, peroxisome proliferator-activated receptor; *SREBP*, sterol regulatory element-binding protein; *TBP*, TATA-box binding protein.

**Table 2:** Food consumption, calorie intake, and body and organ weight.

Parameters	Experimental Groups			
	Control	CDAHFD	Caffeine	CGA
Food consumption (g/day)	3.0 ± 0.2	2.3 ± 0.3 ^a^	2.7 ± 0.4 ^a,b^	2.3 ± 0.3 ^a^
Calorie intake (kcal/day)	10.5 ± 0.5	12.1 ± 1.6 ^a^	14.0 ± 2.0 ^a,b^	12.1 ± 1.3 ^a^
Body weight (g)	25.3 ± 1.4	20.4 ± 0.8 ^a^	20.7 ± 1.2 ^a^	20.6 ± 0.5 ^a^
Liver weight (g)	1.0 ± 0.0	1.3 ± 0.2 ^a^	1.3 ± 0.2 ^a^	1.4 ± 0.0 ^a^
Liver to body weight ratio (%)	3.9 ± 0.3	6.5 ± 0.9 ^a^	6.6 ± 0.2 ^a^	6.9 ± 0.2 ^a^
EAT weight (g)	0.5 ± 0.1	0.3 ± 0.0 ^a^	0.3 ± 0.1 ^a^	0.3 ± 0.0 ^a^
EAT to body weight ratio (%)	2.0 ± 0.2	1.4 ± 0.3 ^a^	1.3 ± 0.3 ^a^	1.5 ± 0.1 ^a^

Data are presented as means ± SD. ^a^ Significantly different from the control group (*p* < 0.05). ^b^ Significantly different from the CDAHFD group (*p* < 0.05). EAT, epididymal adipose tissue.

**Table 3:** Biochemical data of serum.

Parameters	Experimental Groups			
	Control	CDAHFD	Caffeine	CGA
AST (IU/L)	175.3 ± 88.7	326.8 ± 25.1 ^a^	339.6 ± 27.6 ^a^	340.0 ± 61.4 ^a^
ALT (IU/L)	27.7 ± 4.3	272.3 ± 42.3 ^a^	342.4 ± 39.6 ^a,b^	349.7 ± 84.8 ^a,b^
T-Cho (mg/dL)	102.0 ± 3.2	47.3 ± 13.3 ^a^	42.3 ± 6.3 ^a^	55.2 ± 3.6 ^a^
TG (mg/dL)	75.7 ± 23.7	42.7 ± 16.9 ^a^	35.6 ± 2.6 ^a^	48.3 ± 14.1 ^a^
Glucose (mg/dL)	48.3 ± 9.2	65.0 ± 5.8	85.7 ± 40.9 ^a^	78.0 ± 19.9
Insulin (pg/mL)	226.8 ± 134.9	59.2 ± 62.0 ^a^	147.2 ± 125.2	48.2 ± 49.5 ^a^
Adiponectin (µg/mL)	15.4 ± 1.8	12.8 ± 0.2 ^a^	13.1 ± 0.9 ^a^	13.0 ± 1.2 ^a^

Data are presented as means ± SD. ^a^ Significantly different from the control group (*p* < 0.05). ^b^ Significantly different from the CDAHFD group (*p* < 0.05). ALT, alanine aminotransferase; AST, aspartate aminotransferase; T-Cho, total cholesterol; TG, triglyceride.

**Table 4:** Histopathological findings of experimental groups.

Parameters	Experimental Groups			
	Control	CDAHFD	Caffeine	CGA
Steatosis	0 (0, 0)	3 (3, 3) ^a^	3 (3, 3) ^a^	3 (3, 3) ^a^
Intralobular inflammation	0 (0, 0)	3 (3, 3) ^a^	3 (3, 3) ^a^	3 (3, 3) ^a^
Portal inflammation	0 (0, 0)	0.5 (0, 1)	0.5 (0, 1)	0 (0, 1)
Ballooning	0 (0, 0)	1 (1, 2) ^a^	1 (1, 2) ^a^	2 (1, 2) ^a^
NAS	0 (0, 0)	7 (7, 8) ^a^	7 (7, 8) ^a^	8 (7, 8) ^a^
Perisinusoidal fibrosis	0 (0, 1)	2 (2, 2) ^a^	2 (1, 2) ^a^	2 (2, 2) ^a^
Fibrosis stage	0 (0, 1)	1 (1, 1) ^a^	1 (1, 1) ^a^	1 (1, 1) ^a^

Data are presented as median (min., max.). ^a^ Significantly different from the control group (*p* < 0.05). NAS, NAFLD activity score.
